# 

**Published:** 1907-06

**Authors:** 


### THE BRISTOL MEDICO-CHIRURGICAL JOURNAL

LIBRARY
Indexed
S. G. O.

*PUBLISHED UNDER THE AUSPICES OF*

*THE BRISTOL MEDICO-CHIRURGICAL SOCIETY.*

*Editor:* R. SHINGLETON SMITH, M.D., B.Sc.
WITH WHOM ARE ASSOCIATED
J. MICHELL CLARKE, M.A., M.D.,
J. LACY FIRTH, M.S., M.D., JAMES SWAIN, M.S., M.D.
P. WATSON WILLIAMS, M.D., *Assistant Editor.*
*Editorial Secretary:* JAMES TAYLOR.

BRISTOL: J. W. ARROWSMITH.
LONDON: J. & A. CHURCHILL, 7 GREAT MARLBOROUGH STREET.

*Price One Shilling and Sixpence.*

